# Increased Water-Solubility and Maintained Antioxidant Power of Resveratrol by Its Encapsulation in Vitamin E TPGS Micelles: A Potential Nutritional Supplement for Chronic Liver Disease

**DOI:** 10.3390/pharmaceutics13081128

**Published:** 2021-07-23

**Authors:** Guendalina Zuccari, Silvana Alfei, Alessia Zorzoli, Danilo Marimpietri, Federica Turrini, Sara Baldassari, Leonardo Marchitto, Gabriele Caviglioli

**Affiliations:** 1 Department of Pharmacy (DiFAR), University of Genoa, Viale Cembrano 4-I, 16148 Genova, Italy; alfei@difar.unige.it (S.A.); turrini@difar.unige.it (F.T.); baldassari@difar.unige.it (S.B.); caviglioli@difar.unige.it (G.C.); 2 Stem Cell Laboratory and Cell Therapy Center, IRCCS Istituto Giannina Gaslini, Via Gerolamo Gaslini 5, 16147 Genova, Italy; alessiazorzoli@gaslini.org (A.Z.); danilomarimpietri@gaslini.org (D.M.); 3 Department of Sciences for the Quality of Life, University of Bologna, Corso D’Augusto 237, 47921 Rimini, Italy; leonardo.marchitto@unibo.it

**Keywords:** resveratrol, TPGS, drug delivery system, micelles, vitamin E, pediatric formulation, liver disease, nutrition

## Abstract

Children affected by chronic liver disease exhibit impaired neurocognitive development and growth due to the low absorption and digestion of nutrients. Furthermore, malnutrition is an adverse prognostic factor in liver transplantation as it is associated with an increase in morbidity and mortality. D-α-tocopheryl-polyethylene-glycol-succinate (TPGS) is currently administered per os as a vitamin E source to improve children’s survival and well-being; however, TPGS alone does not reverse spinocerebellar degeneration and lipid peroxidation. To potentiate the effects of TPGS, we loaded micelles with resveratrol (RES), a natural polyphenol, with antioxidant and antiinflammatory activities, which has demonstrated protective action in the liver. Firstly, we investigated the suitability of TPGS to encapsulate RES in micelles by means of a phase-solubility study, then RES-TPGS formulations were prepared via solvent casting and solvent diffusion evaporation methods. RES-TPGS colloidal dispersions showed small mean diameters (12 nm), low polydispersity, and quite neutral Zeta potentials. The formulations showed a sustained drug release and a good drug loading capacity, further confirmed by infrared spectroscopy and differential scanning calorimetry. RES-TPGSs exhibited unaltered antioxidant activity compared to pristine RES via the DPPH assay and a significant reduction in toxicity compared to empty TPGS on HaCaT cells. Thus, RES-TPGS micelles may overcome the challenges of current liver disease therapy by providing more protective effects thanks to the antioxidant activity of RES and by reducing the surfactant toxicity on normal cells.

## 1. Introduction

Cholestasis is the final common biochemical phenotype of a variety of congenital and acquired liver pathologies [1]. It can also represent a highly selective impairment of one of many steps involved in the synthesis, secretion, and modification of bile acids, which in turn leads to liver damage. Therefore, cholestatic liver disease comprises a highly heterogeneous group of conditions [2]. In the United States the overall incidence of liver disease in neonates is about 1 in every 2500 newborn babies and at least 45% of cases of cholestatic liver disease in children are genetic in etiology [3]. The primary cholestatic diseases of infancy are symptomatic and often rapidly progressive. These chronic cholestatic diseases include biliary atresia; Alagille syndrome; progressive familial intrahepatic cholestasis diseases (PFIC); bile acid synthesis defects; cystic fibrosis-related liver disease; ductal plate abnormalities, including Caroli syndrome and congenital hepatic fibrosis; primary sclerosing cholangitis (PSC); and certain metabolic diseases. The consequences related to these disorders are malnutritional deficiencies and impaired neurocognitive development. Indeed, children with cholestatic liver disease require an increased energy intake for growth up to 140% of the estimated average requirements, due to their longstanding condition, which progressively reduces glycogen stores, increases protein catabolism and compromises fat absorption. Ultimately, as is known, the normal bioavailability of fat-soluble vitamins is related to fat absorption and requires bile acids and pancreatic enzymes to be present. Therefore, all the conditions in which an insufficient bile amount is secreted lead to impaired vitamin A, D, E, and K absorption, which inevitably deteriorates the health conditions of patients, causing blindness, rickets, neurological morbidities, and coagulopathy, respectively [4]. Notably, if not corrected, vitamin E deficiency may lead to neurological disorders, ranging from peripheral neuropathy to cerebellar ataxia, which becomes irreversible if the vitamin E deficit remains untreated, as the peroxidation of unsaturated fatty acids disrupts the function of central and peripheral nervous system cell membranes. Another consequence of vitamin E malnourishment is hemolytic anemia due to the lack of protection against oxidative damage to erythrocytes’ membranes.

**Figure 1:**
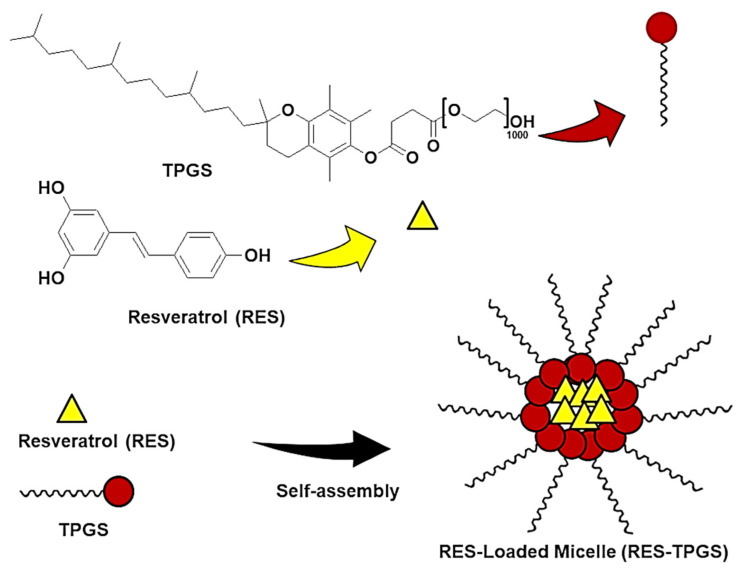
Chemical structures of TPGS and RES and a schematic representation of the micelles.

Concerning the correction of vitamin E deficiency, commercially available vitamin E supplements in Europe rely on parenteral vitamin E or α-tocopherol acetate, but the former is subject to low acceptability by children, and the latter was not found to be sufficiently water-soluble and resulted in low bioavailability [5]. These first formulations were followed by an oral liquid water soluble form (Vedrop^®^), consisting of D-α-tocopheryl polyethylene glycol succinate (TPGS) (Figure 1), approved by the European Medicines Agency (EMA) in 2009, to provide a supplement for patients from birth up to 18 years affected with congenital chronic cholestasis or hereditary chronic cholestasis.

Previously, the scientific advice of the European Food Safety Authority (EFSA) concerning the safety and bioavailability of TPGS as a water-soluble source of vitamin E for use in foods for nutritional purposes was addressed in 2007 [6]. TPGS is formed by esterification of polyethylene glycol 1000 with D-α-tocopheryl via a succinate bridge. According to the EFSA, the recommended TPGS daily intakes administered under medical supervision are 5 mg TPGS/kg bw in teenagers to 13 mg TPGS/kg bw in 1-month-old infants, based on the assumption that each gram of TPGS contains 27% tocopherol. The powder has practically no taste and lacks bitterness; this makes it especially suitable for oral use [7]. TPGS does not require the action of bile salts or pancreatic enzymes for its absorption into the intestinal wall. It can pass freely through cell membranes without competition for transport carriers with other nutrients [6]. In vitro studies have shown that TPGS can be hydrolyzed inside enterocytes to free α-tocopherol [7].

Dietary supplementation is notably important, since many children with chronic liver disease will eventually require liver transplantation, and studies have shown that nutritional deficiencies before and during transplantation are associated with poorer outcomes. Nutritional status is an important prognostic factor, and children with better nutritional status have fewer complications and lower mortality in liver transplantation.

Therefore, nutritional support seems to be one of the priorities and can be regarded as a bridging therapy for liver transplantation [3]. However, the correction of vitamin E deficiency with TPGS may not reverse severe spinocerebellar degeneration, even through intramuscular injection. Moreover, it has been reported that TPGS therapy quickly normalized serum vitamin E levels but did not improve the increased lipid peroxidation and poor polyunsaturated fatty acid levels; therefore, improvements in therapeutic strategies are needed [8].

Here, we tried to find new strategies to enhance the beneficial effects of TPGS and to optimize nutritional support in children with chronic liver disease. Resveratrol (RES) (Figure 1), a natural non-flavonoid polyphenol abundant in grapes, blueberries, and peanuts, possesses diverse biological activities: anti-inflammatory, anti-oxidant, antitumor, and estrogenic [9]; therefore, an association with TPGS could be an optimal one. In preclinical models, RES improved cholestatic liver injury by decreasing fibrosis, oxidative damage, and promoting hepatocyte regeneration by attenuating the release of pro-inflammation cytokines and Kupffer cell accumulation [10]. Its protective role is due to the activation of the farnesoid X receptor (FXR) pathway and the suppression of nuclear factor-*k*B (NF-*k*B) activity [11]. Although several reports have confirmed that RES elicits many health-beneficial effects, its use is limited by its bad pharmacokinetic characteristics, poor solubility, and chemical instability [12]. After oral administration, RES is absorbed in the intestine and undergoes first-pass glucuronidation and sulfate conjugation of the phenolic groups. Its metabolism and low hydro-solubility (0.13 mM) result in in vivo drug concentrations that are unable to provide many of the biological activities emphasized in in vitro experiments [13]. The Biopharmaceutics Classification System (BCS) inserts RES in the second class of drugs, those characterized by low water solubility alongside high intestinal permeability. This classification provides a theoretical basis to correlate in vitro drug dissolution and in vivo bioavailability. Since RES has a limited dissolution rate in the aqueous environment, an increase in solubility may significantly enhance its bioavailability [14]. Moreover, it has been demonstrated that both sulfate and glucuronide derivatives undergo enterohepatic recirculation, which allows their deconjugation in the small intestine and reabsorption, leading to RES accumulation in the target organ, i.e., the liver [15]. In this regard, a delivery system that can facilitate the dissolution of a large amount of RES could effectively increase its plasma concentration and hepatic accumulation. Moreover, in vitro studies demonstrated that an increase in RES solubility determined a partial saturation of its biotransformation to monoglucuronide and mono-sulfate derivatives in Caco-2 cells, with a consequent potential improvement of its bioavailability [16].

In the last decade, to improve RES clinical performance, various methodological approaches have been developed. In detail, the formulations studied include solid lipid nanoparticles (SLNs), liposomes, nano-emulsions, micelles, solid dispersions, polymeric nanoparticles, and cyclodextrins [17]. However, the lipid-based formulations are characterized by low stability and drug loading capacity, whereas polymeric nanoparticles have the drawback of hardly crossing biological membranes and the scarcity of safe polymers represents the main concern [18]. The therapeutic application of solid dispersions is limited by the thermodynamic instability of the amorphous state, whereas cyclodextrins are expensive and not easily scalable [18]. Therefore, a lot of problems persist, such as the long-term safety of nanoparticles and their interaction with biological systems, and researchers have aimed to create reproducible and stable formulations and to improve the RES loading capacity to make them more affordable and easily scalable.

Based on the above, we chose to combine the properties of TPGS as a functional food supplement with its capability to encapsulate lipophilic substances such as RES, with the aim of optimizing nutritional support in children with chronic liver disease. TPGS has been widely studied as an absorption enhancer and emulsifier and it has already been shown to act simultaneously as a nanocarrier and as a permeation enhancer [19]. TPGS is capable of self-assembling in water above its critical micellar concentration (CMC), equal to 0.02 wt %, in nanosized aggregates. It was already reported that TPGS alone improves the oral bioavailability of cyclosporine [20], amprenavir [21], paclitaxel [22], lopinavir [23], curcumin [24], and etoposide [25]. As already mentioned, by containing both a hydrophilic shell and a lipophilic core, TPGS micelles are capable to improve the oral bioavailability of transported drugs, acting simultaneously as a solubilizer, an emulsifier, as well as a permeation and an absorption enhancer. Additionally, TPGS micelles, by protecting the encapsulated drugs from premature degradation in the gastrointestinal (GI) tract, enhance their residence time and systemic concentrations [26]. Here, micelles loaded with RES (RES-TPGSs) were obtained and characterized by Fourier transform infrared spectroscopy (FTIR), thermal analysis (DSC), dynamic light scattering (DLS) for determination of particle size, the polydispersity index (PDI), and Zeta (ζ) potential. In addition, RES-TPGS formulations were assessed for their stability, whereas the in vitro antioxidant activity was determined using the DPPH assay. Finally, RES-TPGS cytotoxicity was evaluated on keratinocyte HaCat cells for the evaluation of the safety of empty and loaded surfactant-based micelles.

## 2. Materials and Methods

### 2.1. Materials

TPGS was a gift from PMC Isochem (Vert Le Petit, France); RES and all reagents were purchased from Merck (formerly Sigma Aldrich, Milano, Italia).

### 2.2. Solubility Studies

The equilibrium (thermodynamic) solubility of RES was determined using the shake-flask method. Briefly, assays were performed in flasks with a capacity of 10 mL. A weighed drug amount, sufficient to saturate the solution, was added to different surfactant solutions and maintained at 37 °C ± 0.2 °C in a water bath (RCS 6 Lauda Thermostat) under vigorous stirring. TPGS concentrations ranged from 0.05 to 3.30 mM. Samples were kept in the dark and allowed to equilibrate until RES concentration in the solution remained constant. After 48 h under stirring, the samples were filtered using a 0.45-µm filter (Minisart RC Sartorius, Göttingen, Germany). Aliquots of each filtrate were diluted with methanol to disrupt the micelles and were analyzed using an UV-Vis spectrophotometer (HP 8453, Hewlett Packard, Palo Alto, CA, USA) at λ_max_ = 306 nm. All solubilities were measured in triplicate and reported as the mean ± the standard deviation (SD) of the mean versus TPGS concentrations. TPGS solutions alone were used as blanks. The total RES solubility was calculated using a previously constructed standard calibration curve.

### 2.3. Formulations Preparation

#### 2.3.1. Solvent Diffusion Evaporation Method

This method involves dissolving the surfactant and the drug in an organic water-miscible volatile solvent [27]. To screen for a suitable TPGS amount that entirely entrapped RES, different drug/surfactant ratios were assayed. Briefly, a fixed amount of RES (20 mg) was solubilized in acetone (1 mL), containing increasing TPGS amounts (1:1, 1:2, 1:3, 1:4, 1:5, 1:6, 1:7; *w*/*w*). Subsequently, the organic solution was added dropwise in 10 mL Milli-Q (m-Q) water at room temperature under vigorous stirring and stirred for 1 h to allow solvent evaporation. Finally, the samples were filtered through a 0.22-µm filter to remove the excess non-encapsulated drug and used for further characterizations. Control formulations were prepared as above without RES.

#### 2.3.2. Solution Casting Method

RES micelles were also prepared using the solution casting method, according to the previously published procedure with slight modifications [19]. The same preparative mixtures described in the previous subsection were employed and solubilized in 6 mL of ethanol; in the case of blanks, only TPGS was included. Then the solvent was evaporated under a vacuum at 40 °C using a rotavapor and the melted mixture was cooled to room temperature to obtain drug dispersion into a TPGS thin-layer film. Subsequently, the dried film was hydrated in an orbital shaker at room temperature for 1 h using 10 mL of m-Q water. After filtration, all samples were further characterized and compared.

### 2.4. Determination of the Encapsulation Efficiency and Drug Loading Capacity

The amount of RES in the freshly prepared colloidal dispersions was spectrophotometrically measured after dilution in methanol as previously described and the drug entrapment efficiency percentage (EE%) was calculated according to the formula in Equation (1).

(1) $$\mathrm{EE}\%=\frac{\mathrm{Drug}\;\mathrm{wt}\;\mathrm{in}\;\mathrm{the}\;\mathrm{filtered}\;\mathrm{micellar}\;\mathrm{colloidal}\;\mathrm{dispersion}}{\mathrm{Drug}\;\mathrm{wt}\;\mathrm{added}\;\mathrm{in}\;\mathrm{the}\;\mathrm{preparative}\;\mathrm{mixture}}\times 100$$

Afterwards, the freshly prepared micellar dispersion was aliquoted in 2-mL samples and frozen at −20 °C. After 24 h, the vials were freeze-dried (Labconco, Kansas City, MI, USA). The temperature of the lyophilization chamber was set at −30 °C and that of the condenser was −40 °C. After thermal equilibration of the samples with the chamber temperature, indicated by a thermal probe inserted and congealed within a solution of a control sample, sublimation was performed by reducing the pressure up to 20 × 10^−3^ mbar. After 48 h, the secondary drying process was performed, raising the temperature up to 25 °C for 1 h. The freeze-dried micelles were stored in a dryer until further use.

The drug loading capacity (DL%) was determined according to the formula in Equation (2), starting with a weighed lyophilized RES-TPGS powder sample reduced by TPGS CMC to consider only the fraction of TPGS forming the micelles and assuming in both equations that the amount of free drug in the solution was negligible, owing to its poor solubility in water.

(2) $$\mathrm{DL}\%=\frac{\mathrm{wt}\;\mathrm{of}\;\mathrm{entrapped}\;\mathrm{drug}}{\mathrm{wt}\;\mathrm{of}\;\mathrm{the}\;\mathrm{loaded}\;\mathrm{micelles}}\times 100$$

### 2.5. Determination of Micelle Size, Polydispersity Index (PDI), and Zeta (ƺ) Potential

The particle size (Z-average), polydispersity index (PDI), and Zeta potential (ƺ) of colloidal preparations were measured at 25 °C using a Malvern Nano ZS90 light scattering apparatus (Malvern Instruments Ltd., Worcestershire, UK) at a scattering angle of 90°. The apparent equivalent hydrodynamic radii of the micelles were calculated using the Stokes–Einstein equation. The ƺ potential values of micelles were recorded with the same apparatus, in distilled water at 25 °C. The results from these light scattering experiments were presented as the average values ± SD obtained from three different batches and carrying out three runs of ten measurements per sample.

#### Re-Dispersibility Study of Lyophilized RES-TPGS Micelles

The reconstitution of lyophilized RES-TPGS micelles was evaluated according to a previously published method [28]. m-Q water was added to the dried powders up to the same concentration as that prior to the freeze-drying process. The samples were rehydrated for 2 min and then vortexed for 30 s. Subsequently, the particle size and PDI were measured as described above. Afterwards, the average value of the measurements was calculated and used to determine the ratio of mean particle size (*S_l_)* after the reconstitution of freeze-dried samples and the initial mean particle size (*S_f_*) of the fresh samples. The results are expressed as the particle size ratio (*S_l_*/*S_f_*) according to Equation (3).

(3) $$\frac{S_{l}}{S_{f}}=\frac{\mathrm{mean}\;\mathrm{particle}\;\mathrm{size}\;\mathrm{of}\;\mathrm{lyophilized}\;\mathrm{micelles}}{\mathrm{mean}\;\mathrm{particle}\;\mathrm{size}\;\mathrm{of}\;\mathrm{fresh}\;\mathrm{micelles}}$$

### 2.6. Principal Component Analysis (PCA)-Assisted FTIR Spectroscopy

FTIR analyses of RES, TPGS, and RES-TPGSs at 1:2, 1:3, 1:4, 1:5, 1:6, and 1:7 *w*/*w* were performed by formulating the samples in KBr pellets. The FTIR spectra were recorded on a Spectrum Two FT-IR Spectrometer (PerkinElmer, Inc., Waltham, MA, USA). The spectra were acquired in triplicates for each compound in transmission mode and both in the transmittance and absorbance scale. Acquisition was made from 4000 to 500 cm^−1^, with 1 cm^−1^ spectral resolution, co-adding 32 interferograms, with a measurement accuracy in the frequency data at each measured point of 0.01 cm^−1^, due to the internal reference laser of the instrument. The frequency of each band was obtained automatically by using the “find peaks” command of the instrument software. The matrix of spectral data was subjected to PCA using R statistical software, which was freely downloadable (http://cran.mirror.garr.it/mirrors/CRAN/—Garr Mirror, Milan, Italy, accessed on 22 July 2021).

#### Chemometric Analysis: PCA

To confirm the presence of RES in TPGS micelles, we processed the FTIR data sets of the most significant spectra in *n* measurable variables Therefore, we excluded the FTIR spectra of samples 1:4 and 1:5, and considered those of samples that, according to the DL% results, contained the highest and lowest concentrations of RES. For each sample, the variables consisted of the values of absorbance (%) associated with the wavenumbers (3501) in the range 4000–500 cm^−1^. To obtain reliable information, we exploited PCA, which reduced the large number of variables to a small number of new variables, namely, principal components (PCs). PCA was performed on a matrix of data of 8 × 3501, including a total of 28,008 variables. Spectral data were preprocessed using the standard normal variate (SNV) transform to minimize global intensity effects due to slightly different optical paths and via column mean-centering.

### 2.7. Differential Scanning Calorimetry (DSC)

To confirm the entrapment of RES inside the micelles, DSC analysis was performed. The thermal properties of lyophilized RES-TPGSs, free RES, and of physical mixtures of the raw RES and TPGS in equal ratios to those in the prepared RES-TPGSs were studied using a DSC-7, equipped with Pyris software (PerkinElmer, Inc., Waltham, MA, USA). The instrument was calibrated with Indium and Zinc and about 2.5 mg of samples were crimped in aluminum pans. The thermograms were recorded from 10 °C to 285 °C at a heating rate of 10 °C/min under nitrogen flow.

### 2.8. Formulation Stability

The stability of micelles is critical to protect the entrapped hydrophobic component in the core. For micelles, stability is generally defined in terms of thermodynamic as well as kinetic stability. Thermodynamic stability is achieved when the surfactant concentration is above the CMC. However, micelles are a dynamic system, and the exchange rate of amphiphilic unimers between the bulk and the micelles may determine their disassembly. RES-TPGSs were made and stored in the liquid state prior to freeze-dry processing. Therefore, the stability of the micelles was investigated by storing the colloidal dispersions in the liquid state at 25 °C in a WTB © BINDER GmbH 2015–2020 (Im Mittleren Ösch 5, D-78532 Tuttlingen, Germany) incubator and visually observed after 24, 48, and 72 h for signs of precipitation. At each time point, the drug concentration in the solution was determined after filtration to remove RES leakage. The determinations were made in triplicate and results were reported as mean ± SD.

### 2.9. In Vitro Release Studies

The in vitro release of RES from the optimized formulation was performed via the dialysis method [29] at 37 °C. An amount of RES-TPGS lyophilized powder in a 1:5 formulation, corresponding to 2 mg of RES, was reconstituted with 5 mL of phosphate buffer solution (PBS) at pH 7.4 and filled in a dialysis tube (MW CO 12,000 Da, Spectrum^TM^, Spectra/Pore^®^, USA). A corresponding RES raw powder was suspended in the same volume to obtain an equal drug concentration and was tested along with the loaded micelles. Then, the bags loading RES preparations were dialyzed against 200 mL of PBS at 37 °C, in which 1% (*v*/*v*) Tween 80 was added to assure sink conditions. Each assay was performed in triplicate, including a negative control of PBS with TPGS alone [23]. The experiment was carried out under continuous stirring and 2 mL of dialysate was withdrawn at different time points for the determination of the drug concentration as described above. Samples were diluted to the calibration range with methanol. An equal volume of fresh buffer was immediately replaced after every sampling. The cumulative release was calculated according to Equation (4):

(4) $$\mathrm{Cumulative}\;\mathrm{release}\;\%=\frac{M_{t}}{M_{\infty }}\times 100\%$$

where *M_t_* is the total amount of RES that had been released in the medium, including the amount being sampled at every time point, and *M_∞_* is the initial RES amount in the dialysis tube. The release study was performed in triplicate, and the cumulative percentage of RES released was represented as the average ± S.D.

The data obtained from the drug release study were applied to different release kinetics models such as the Higuchi, zero-order, first-order, Korsmeyer–Peppas, and Hixson–Crowell models to predict the drug’s release mechanism. The fit model with the highest correlation coefficient (*R*^2^) value was considered the best.

### 2.10. Determination of the Radical Scavenging Activity (RSA) of RES-TPGSs

The antioxidant activity of the samples was screened using the in vitro DPPH·(1,1-diphenyl-2-picryl-hydrazyl) assay [30]. Briefly, 250 μL of each sample were transferred into a 10-mL volumetric flask and mixed with a daily-prepared 10^−4^ M DPPH methanol solution. After 30 min incubation in the dark and at room temperature, the residual absorbance was read at 515 nm against a blank (solution without radical). The initial DPPH concentration was measured using control samples obtained by diluting 250 μL of methanol with the DPPH solution in a 10-mL volumetric flask. Four samples were employed: the RES raw powder (dilution 1:10 *w/v* in methanol), the RES lyophilized raw powder (dilution 1:10 *w/v* in methanol), and two RES-TPGS formulations (1:2 and 1:6 ratios) diluted in methanol according to DL%, resulting in an RES concentration equal to 0.1 mg/mL in all samples. TPGS alone was also tested at the concentration of 0.90 mg/mL. The absorbance measurements were transformed in free radical scavenging activity (% RSA, or percent antioxidant activity) according to Equation (5):

% RSA = [(A Control − A sample)/A Control] × 100(5)

The percentage of DPPH inhibition (decrease in absorbance) was calculated considering DPPH alone as 100%: the lower the absorbance, the higher the antioxidant power. For each sample, two replicated determinations were performed, and results were reported as mean ± standard deviation.

### 2.11. Cytotoxicity Evaluations

#### 2.11.1. Cell Culture

Human skin keratinocyte cells (HaCaT) were grown as a monolayer in RPMI 1640 medium supplemented with 10% fetal bovine serum (*v*/*v*), 1% penicillin-streptomycin, 1% glutamine (Euroclone S.p.A., Milan, Italy), cultured in T-25 cm^2^ plastic flasks (Corning Incorporated (NYSE:GLW), Corning, NY, USA) and maintained at 37 °C in 5% CO_2_ humidified atmosphere. Cells were tested and characterized at the time of experimentation, as previously described [31].

#### 2.11.2. Viability Assay

Immortalized human keratinocyte HaCaT cells were seeded in 96-well plates at 4 × 10^3^ cells/well in 200 μL complete medium and cultured for 24 h. The seeding medium was removed and replaced with 100 μL fresh complete medium that had been supplemented with increasing concentrations of free or encapsulated RES (2.5 μM, 5 μM, 10 μM, 20 μM, 40 μM) or with the corresponding concentration of empty TPGS micelles (2.01 μM, 4.02 μM, 8.04 μM, 16.07 μM, 32.15 μM), according to the sample DL%. Cells were then incubated for an additional 12 or 24 h, after which the effect on cell growth was evaluated using the fluorescence-based proliferation and cytotoxicity assay, the CyQUANT^®^ Direct Cell Proliferation Assay (Thermo Fisher Scientific, Life Technologies, Monza Brianza, Italy), according to the manufacturer’s instructions. Briefly, at the selected times an equal volume of detection reagent was added to the cells in culture and incubated for 60 min at 37 °C. The fluorescence of the samples was measured using a monochromator-based M200 plate reader (Tecan, Männedorf, Switzerland) set at 480/535 nm. The experiments were carried out at least three times and each sample was run in quadruplicate.

### 2.12. Statistical Analyses

To assess the existence of statistical differences between the mean of one sample and the mean of the population a one-sample *t*-test (PAST: paleontological statistics software package for education and data analysis, free downloadable online, at: https://past.en.lo4d.com/windows, accessed on 22 July 2021) was employed. Concerning biological studies, the statistical significance of differences between experimental and control groups was determined via a two-way analysis of variance (ANOVA) with the Bonferroni correction. The analyses were performed with Prism 5 software (GraphPad, La Jolla, CA, USA). Asterisks indicate the following *p*-value ranges: * = *p* < 0.05, ** = *p* < 0.01, *** = *p* < 0.001.

## 3. Results and Discussion

### 3.1. TPGS Suitability for RES Encapsulation

We investigated the great potential of TPGS to play two key roles simultaneously, as a nanocarrier and as a vitamin E source. Recently, some preclinical studies have highlighted the preventive role of RES in liver disease [32], but a formulation that is able to comprise the two active ingredients has not been developed yet. For this purpose, we first evaluated the capability of TPGS to encapsulate RES, performing phase solubility studies by applying TPGS concentrations below and above its CMC value (0.2 wt %, 0.132 mM) [33]. These studies involve the formation of micelles using the equilibrium method, which is a direct dissolution method, with the surfactant and the guest molecule simply added in a water solution. In this context, solubilization can be defined as the spontaneous dissolution in water of a molecule by reversible interactions with the micelles to form a thermodynamically stable isotropic solution with reduced thermodynamic activity of the solubilized material.

The concentration of RES at equilibrium was determined by UV-Vis spectrophotometric analysis using a constructed linear calibration curve, Equation (6), (Figure S1 in the Supplementary Information (SI)). The high value of the correlation coefficient (*R*^2^ = 0.997) related to the curve assured linearity within a RES concentration range of 3.33–331.70 µM.

(6) y=128.51x+0.03

**Figure 2:**
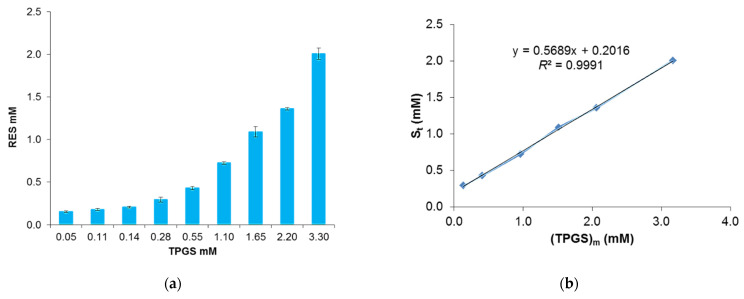
TPGS concentration-dependent solubility of RES. Total solubility of RES versus TPGS concentration, measured using the equilibrium method after 48 h at 37 °C in water (**a**); linear regression obtained considering the values above the TPGS CMC (**b**).

This solubilizing method is characterized by low drug encapsulation efficiency, due to the very low solubility of RES, but it is indicative of the potential of TPGS to act as a good host for the guest molecule. According to the *European Pharmacopeia*, RES is defined as “practically insoluble” in water as its hydro-solubility is near 3 mg/100 mL (0.13 mM). Indeed, RES is a fat-soluble compound, soluble in ethanol at about 50 mg/mL (200 mM) and in DMSO at 16 mg/mL (70 mM). As shown in Figure 2a, RES’s total solubility (namely free plus encapsulated RES) is directly proportional to the TPGS concentration, indicating the distribution of RES into the micelles.

Note that the formation of micelles causes the RES concentration to increase comparatively rapidly above TPGS 0.14 mM. Thus, this TPGS concentration, above which an abrupt change in the value of total RES solubility occurred, may be considered the CMC. This proportional relation can be seen in Figure 2b, indicating that solubilization is related to micellization. At the highest TPGS concentration tested, RES solubility reached the value of 2 mM, which corresponds to a 15-fold increase. On the contrary, the slight increase in RES solubility below TPGS 0.14 mM is probably due to the presence of weak interactions between the drug and the TPGS unimer.

The estimation of the equilibrium constant (*K_a_*) for the free form of the drug and the encapsulated drug can be deduced using Equations (7)–(9):

(7) $$S_{tot}=S_{free}+S_{bound}$$

(8) $$K_{a}=\frac{S_{bound}}{S_{free}\times {\left(\mathrm{TPGS}\right)}_{m}}$$

(9) $$S_{tot}=S_{free}\;[1+K_{a}\left(\mathrm{TPGS}{)}_{m}\right]$$

where (TPGS)*_m_* is the TPGS concentration forming micelles, which corresponds to the difference in the apparent TPGS concentration and its CMC, (*S_free_*) is the free RES concentration, (*S_tot_*) is the total RES concentration, and (*S_bound_*) is the RES associated to TPGS. The free drug concentration was estimated to be equal to RES solubility in water. The linear regression obtained by plotting *S_tot_* versus (TPGS)*_m_* with Equation (9) led to a *K_a_* value of 4.38 mM^−1^, obtained by dividing the slope by *S_free_* (Figure 2b). This value is higher in comparison with other previous TPGS-based formulations loaded with paclitaxel and estradiol (0.86 mM^−1^ and 0.22 mM^−1^, respectively) [22,34], confirming the suitability of TPGS as an encapsulating agent for RES.

### 3.2. Preparation and Characterization of RES-TPGS Formulations

In the pre-formulation phase, the drug-to-surfactant ratio was varied in order to determine the best encapsulation conditions, and two preparative techniques were also performed for the preparation of RES-TPGS. In both cases, micellization occurred at room temperature (r.t.), as it is well known that temperature greatly influences this process. Indeed, the amount of drug solubilized by micellization increases along with the temperature rise, due to the increase in thermal agitation, which leads to a larger space available inside the micelle, in addition to the increase of the drug’s solubility in water. However, we preferred to perform drug internalization at r.t. in order to avoid supersaturated solutions, which can undergo rapid massive precipitation.

**Figure 3:**
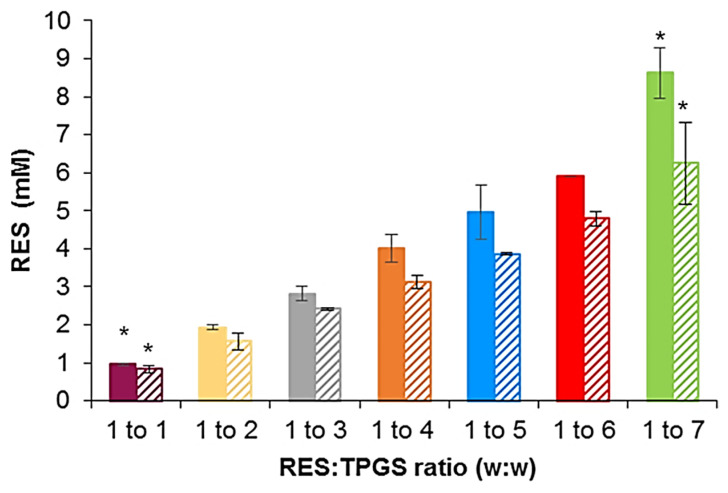
RES total solubility obtained via the solvent casting method and the solvent diffusion evaporation method (striped columns) as a function of the RES:TPGS w:w ratio present in the starting mixture. Histogram summarizing the quantitative data of the means ± S.D. of six independent experiments. * Statistically significant difference vs. formulations prepared by the same method but containing different TPGS concentrations. No statistically significant differences were observed between formulations having the same polymer concentration but prepared using different methods (F-test (ANOVA) and *t*-test, α = 0.05, degree of freedom = 2).

The solution casting method is the most widely employed method since it involves the dissolution of both the drug and polymer in an organic solvent. The only limitation is to find a solvent, avoiding the chlorinated ones, that is able to solubilize both ingredients. Here, we used ethanol, and the thin film obtained after solvent evaporation contained drug molecules tightly entangled with TPGS, so that, when reconstitution in water occurred, the self-assembling driven force of TPGS, allowed for drug encapsulation inside the micelle core. In this case, the drug was encapsulated in the micelles during their formation and not in a second step. Regarding the solvent diffusion evaporation method, when the water-miscible organic solvent diffused in the bulk, TPGS still remained dissolved; on the contrary, RES was mostly precipitated. Indeed, we noticed that the aqueous solution turned opalescent after the addition of the organic phase, and then in only some cases, depending on the TPGS concentration, became more transparent after a few minutes. Therefore, in this method, RES encapsulation probably occurred later or after micelle formation. As reported in Figure 3, the casting method was confirmed as the most successful, leading to higher RES total solubility at every tested ratio. We also investigated if the stirring time could enhance RES entrapment in the solvent diffusion evaporation method, by selecting more prolonged times (2 and 3 h), but no significant differences were observed.

The measurements of total solubility showed that the amount of RES in the solution increased according to the addition of TPGS from 0.96 ± 0.02 mM to 8.6 ± 0.67 mM and from 0.84 ± 0.10 mM to 6.25 ± 1.07 mM for the solvent casting and solvent diffusion evaporation methods, respectively. Indeed, by increasing the surfactant concentration, a greater number of micelles was established; thus, more RES molecules could be encapsulated in the inner core of the micelles. The 1:7 formulations were able to incorporate enough RES to increase the total drug content in the solution by about 66-fold and 48-fold. This enhancement in solubility was higher compared to others obtained by different delivery systems such as nano-sponges by about 12-fold [35], and solid dispersions by 5.5-fold [36]. Unfortunately, most research studies did not report these data; therefore, a comparison is rarely possible [37]. Hence, considering their formation through simple self-assembly in solution, RES-TPGS micelles were characterized by their easier preparation and higher scalability in comparison to other nanocarriers, such as liposomes, which require expensive manufacturing processes. The RES encapsulation efficiency (EE%) and the drug loading capacity (DL%) associated to each formulation prepared using the solution casting method are reported in Table 1.

The data confirmed the suitability of TPGS and of the preparative method as a good EE and DL were achieved. The drug’s localization inside the micelles strictly depends on its polarity. RES has a log P of 3.1 but it displays three –OH groups. As for other hydrophobic molecules, the amount of drug loading is greatly influenced by the number of hydrophobic interactions occurring between the guest molecule and the lipophilic core, and these interactions seem to prevail, given the results obtained.

In addition, in this study, the concentration of TPGS providing high encapsulation efficiency is extraordinarily low. Indeed, the TPGS amounts used for RES encapsulation ranged from 2 mg/mL to 14 mg/mL (0.2–1.4 *w*/*v*%), which is far lower compared to those suitable for retinoic acid encapsulation [19], etoposide [25], although it is comparable to the effect of TPGS on the solubility of lopinavir [23] and paclitaxel [22].

Regarding the micellar size, its role in biodistribution is well described in the literature. It manages carrier diffusion across the mucus layer or the uptake into epithelial cells [29]. The mean diameters of the freshly prepared formulations of the main peaks were found to be 11.4 nm and 14.3 nm for the loaded and the empty micelles, respectively, calculated via the intensity-based analysis. It was found that intensity percent analysis is more suitable for aggregation analysis, whereas number-based analysis was more suited for the determination of hydrodynamic radius, since the first one is proportional to the sixth power of particle radius and number-based analysis can normalize this factor. Therefore, in the intensity-based distribution, if aggregation occurs, it could be determined more easily than in the volume- and number-based distributions because the intensity of the scattered light is calculated to be proportional to the 6th power of the particle size [38]. For this reason, we reported the size measurement regarding both the fresh and the reconstituted formulations via intensity-based distribution (Table 2, Figure S2).

As reported, the mean hydrodynamic diameter of empty micelles is slightly larger compared to the loaded ones, suggesting that the RES encapsulation induces core packing and dehydration. This trend is evident also for the loaded micelles, where the formulation with the highest drug loading is characterized by the smallest sizes. It is argued that hydrophobic interactions are augmented during drug encapsulation, and this sometimes triggers contraction of the core, as previously reported in the TPGS-carbamazepine system [39]. Concerning the Zeta potential, since TPGS does not possess ionizable groups, its surface charge is almost neutral (Figure S3). In agreement with other results on other PEGylated delivery systems, a hydrophilic and neutral surface enhances mucus penetration in oral formulations and avoids the formation of protein corona, increasing the circulation time in the blood stream, when intravenously injected [29,40]. Size distribution analysis was also performed on RES-TPGS formulations after freeze-drying. No statistical differences were detected between the fresh and the lyophilized formulations, and only a slightly increase in PDI was observed, probably associated to their aggregation tendency induced by the loss of water during drying. The *S_l_*/*S_f_* ratio never exceeded 1.3 ± 0.4 (Table S1). Values of *S_l_*/*S_f_*~1.0 ± 0.3 indicate that the initial size is maintained, whereas larger values approaching 2 or more suggest a significant aggregation of nanoparticles [41]. Indeed, as recently reported, these systems may not need any cryoprotectant adjunct, as already demonstrated in previous works [19,42].

### 3.3. FTIR Analysis Assisted by PCA

FTIR allows us to quickly achieve chemical information about manufacturing processes, sample chemical structure and quality, as well as differences or similarities in chemical composition of analyzed samples [43]. In this work, the FTIR analyses were carried out on individual components (RES and TPGS), and on lyophilized RES-TPGSs having different RES:TPGS (*w*/*w*) ratios, to qualitatively evaluate the success of the encapsulation reaction.

#### 3.3.1. FTIR Spectra

**Figure 4:**
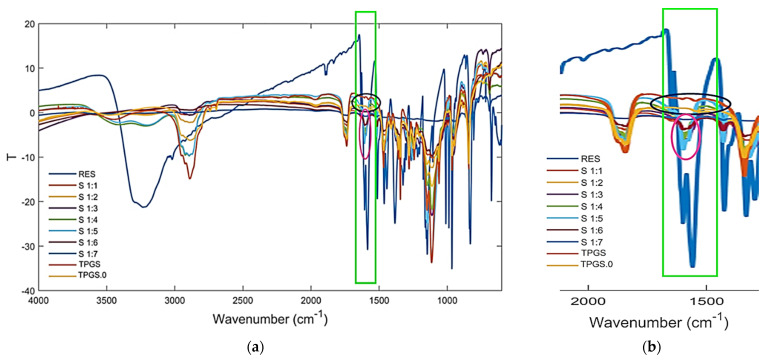
Fourier transform infrared spectroscopy (FTIR) spectra of RES, two different samples of TPGS and of the RES-TPGS formulations prepared (**a**); magnification of a significant spectral region (**b**).

Figure 4a shows the FTIR spectra of RES, TPGS, and of RES-TPGS samples containing RES and TPGS in 1:1–1:7 (*w*/*w*) ratios, whereas Figure 4b shows a significant region of the spectra of the formulations, where typical signals of RES are detectable.

In particular, RES (blue spectrum) has typical bands at 1585, 1607, and 1633 cm^−1^ (light green rectangles), which are missing in the spectrum of TPGS (dark blue horizontal ovals), though they were clearly detectable in several formulations, including formulations with RES:TPGS ratios from 1:3 to 1:7 (fuchsia vertical ovals). Furthermore, in the same region, it can be observed that in all formulations, a peculiar band of TPGS (1739 cm^−1^), absent in the spectrum of RES, was undoubtedly detectable. Consequently, by means of a simple observation, the FTIR spectra established that encapsulations were successful and that all formulations unequivocally contained both RES and TPGS. However, to confirm this empirical assumption in a more reliable way, we performed principal components analysis (PCA), which is a widely used chemometric analytical tool. PCA, working on the complex matrix of spectral data, allows researchers to identify, among the sample population, clusters providing useful information concerning the composition of formulations on the basis of their position in the plot (scores plot).

#### 3.3.2. Principal Component Analysis (PCA)

**Figure 5:**
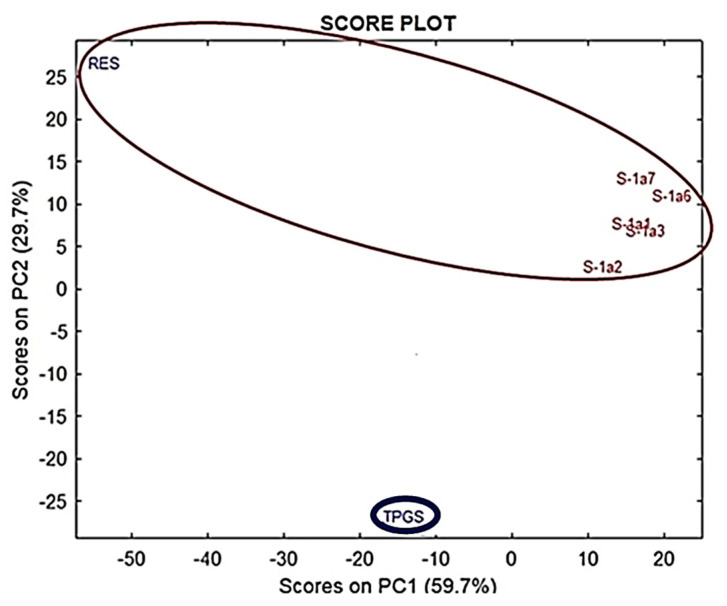
Principle component analysis (PCA) results, represented as a score plot, performed on the matrix collecting spectral data of RES, TPGS, and 1:1–1:3 and 1:6–1:7 RES-TPGSs prepared using R software.

PCA is a method that is widely used in multivariate analysis (MVA) to process spectral data consisting of thousands of variables that require data reductions. The new variables, reduced in number, are called principal components (PCs). PCs are orthogonal linear combinations of the original variables that efficiently represent data variability in low dimensions. The information provided by PCs is expressed as a percentage of the explained variance [44,45]. PCA provides score plots, where one component (e.g., PC1) is displayed vs. another (e.g., PC2), and where the samples under study assume specific positions (scores), forming groups of similar compounds. The position taken by each sample on the selected component can give predictive information on its chemical composition. The results of PCA allowed us to cluster the analyzed samples, and to visualize their positions in relation to those of pure RES and TPGS. Figure 5 shows the PCA results, represented as a score plot.

Accordingly, all the RES-TPGS formulations processed were located more distantly from RES than from TPGS in PC1, indicating that the more abundant ingredient in micelles was TPGS. On the other hand, in PC2, all formulations exhibited score plots that were positive for RES, thus indicating a significant contribution of RES in their composition.

### 3.4. Thermal Analysis Using Differential Scanning Calorimetry (DSC)

**Figure 6:**
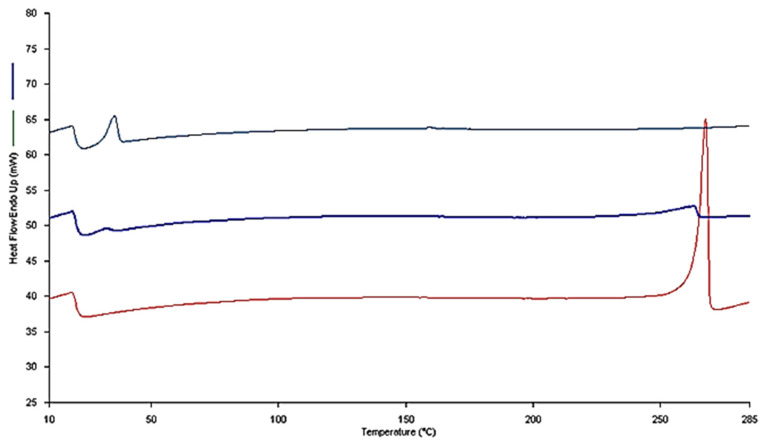
DSC thermograms of a selected RES-TPGS 1:4 formulation (gray line), RES:TPGS 1:4 physical mixture (blue line), and raw RES (red line).

DSC thermograms of lyophilized drug-loaded formulations with a ratio of RES:TPGS of 1:4 (w:w), as representative micellar compounds of all those prepared, in comparison to the corresponding physical mixture, as well as to pure RES, are depicted in Figure 6. The RES thermogram shows the characteristic endothermic peak at 268 °C due to RES’s melting point. TPGS shows an endothermic peak at 32 °C [19]. The DSC thermograms of the RES-TPGS lyophilized formulation showed only the melting peak of the TPGS, indicating the absence of the drug in the crystalline state, and suggesting that the encapsulated drug was in an amorphous state of a solid molecular dispersion into the TPGS core. Furthermore, the physical mixture scan appeared remarkably different, showing both the endothermic peak of TPGS, and a broad peak at 264 °C of RES, thus confirming the presence of the melting peaks of the raw materials.

### 3.5. Stability of RES-TPGSs

**Figure 7:**
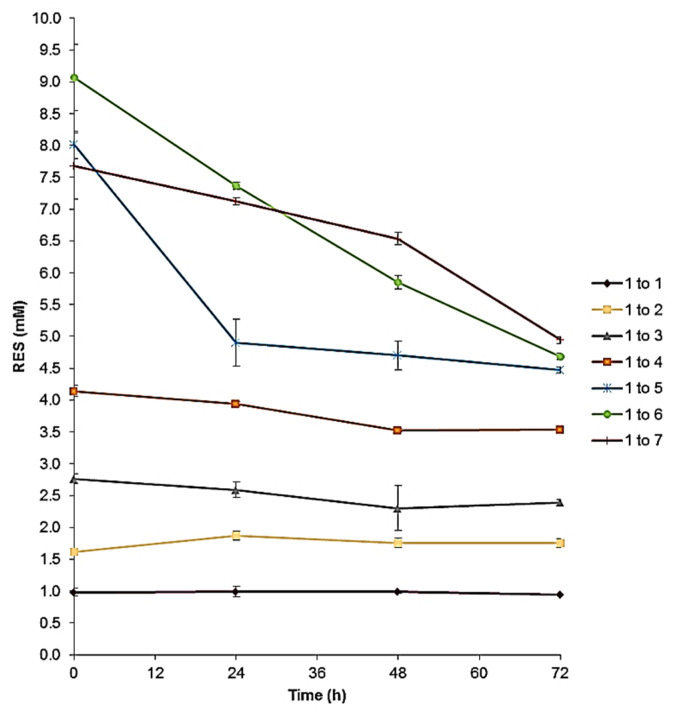
Stability over time of aqueous micellar dispersions maintained at 25 °C, obtained using RES:TPGS ratios of 1:1, 1:2, 1:3, 1:4, 1:5, 1:6, and 1:7 (*w*/*w*) in the preparative mixture.

To investigate if the initial drug loading could affect the performances of the micelles prepared via solvent casting, a stability study was carried out on freshly prepared micelle dispersions. Indeed, to better identify which formulation among the seven would be more valuable for further investigations, stability studies were performed by keeping RES-TPGS colloidal dispersions in an incubator at 25 °C to simulate shelf conditions. In this regard, the detection of total RES in the solution was measured after 24, 48, and 72 h (Figure 7).

The major danger associated with these delivery systems is represented by the tendencies of micelles towards deformation and disassembly, which may lead to leakage and the burst release of the loaded drug. Note that, as long as RES remained in the micellar core, the colloidal dispersion denoted stability and appeared clear; on the contrary, when instability and RES leakage occurred, turbidity and precipitation were observed. As shown in Figure 7, the samples with 1:5, 1:6, 1:7 RES:TPGS ratios, characterized by the highest drug loading, displayed major instability, with extensive RES precipitation over time, evident at the bottom of the tube. It can be speculated that at higher TPGS concentrations, RES supersaturation during hydration occurred, leading to extensive drug precipitation over time. Another explanation may rely on the variation of the hydrophilic and hydrophobic balance upon increased loading of the lipophilic moiety (RES) into the core region, which may trigger the decreased stability of the micelles. On the contrary, the samples in which the percentage of drug loading was lower (1:1, 1:2, 1:3, 1:4) were characterized by more constant maintenance of the RES concentration.

### 3.6. In Vitro Drug Release Studies

**Figure 8:**
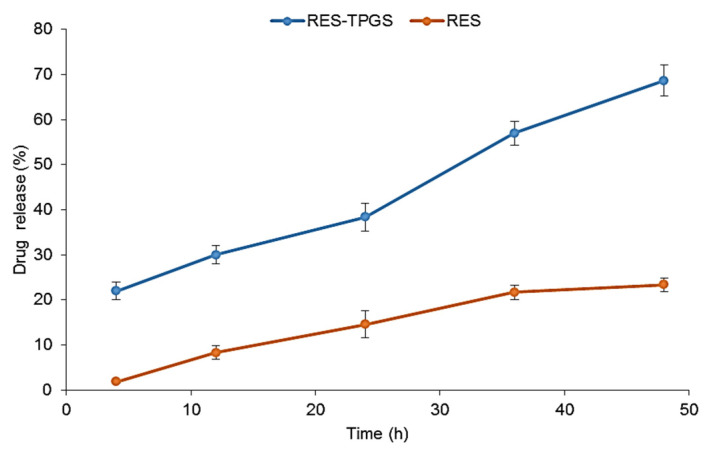
In vitro drug release % over time from the RES:TPGS 1:4 (*w*/*w*) formulation and an RES suspension in PBS at 37 °C.

Taking the above results into consideration, the RES-TPGS formulation prepared using an RES:TPGS ratio of 1:4 (*w*/*w*) in the starting mixture was selected for use in the drug release experiments, as, among the most stable formulations, it was endowed with the highest EE% and DL% values. The encapsulation of drugs in supramolecular systems aims primarily to provide the controlled release of the active ingredients. Here, the RES-TPGS formulation exhibited a sustained release profile, in which the cumulative release percentages were about 30% within 12 h, and about 40% after 24 h in phosphate-buffered saline (pH = 7.4) (Figure 8). This release behavior could be explained through the location of the RES in the micelles and reflected the stability of the RES incorporation. An initial burst happened to a small extent within the first 4 h, and this may be mainly attributed to the drug located in the hydrophilic corona of micelles. Thereafter, the slower RES release resulted from the drug localized in the inner core of micelles. The improvement in the dissolution profile of RES-TPGS is due to the availability of a larger surface area of nano-sized micelles in comparison to the raw control RES suspension. For applications requiring per os administrations of TPGS, hydrolytic stability becomes an important issue. Since TPGS is an ester, it can be expected to undergo hydrolysis of the ester linkage after prolonged exposure to strong acidic or alkaline pH values. According to the manufacturer’s instructions, in an aqueous environment TPGS is stable at pH 4.0, 6.8, and 8.1 for up to 90 days. However, it degrades in an alkaline environment, whereas its stability is still high even at lower pH, and 3.4% of TPGS degraded within 8 h at pH 1.0 and 37 °C [46]. Here, we performed a release study at physiological pH to undertake a comparison between release studies and toxicity studies.

**Figure 9:**
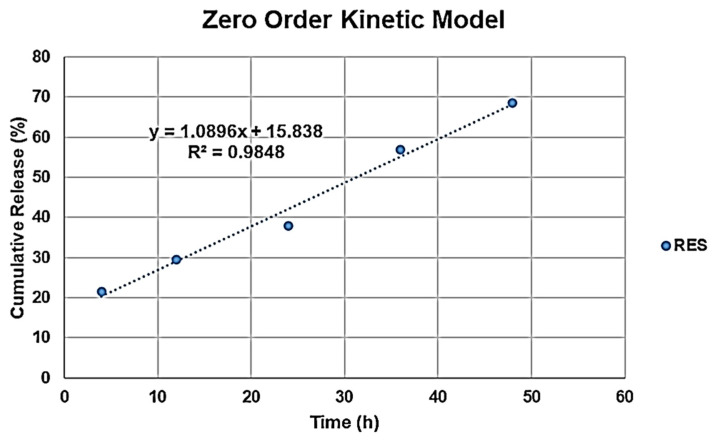
Zero order kinetic mathematical model.

In all cases of supramolecular systems, it is impossible to find a model which simultaneously takes into consideration the structure and properties of the carrier, as well as the drug features and the interactions which may be established among each other. Indeed, different mechanisms can govern the drug release from micelles, including micelle relaxation, unimer dissociation, and molecular diffusion. Therefore, if the experimental data fit well in a model, this is considered applicable. To establish which process occurred in our case, different kinetic mathematical models were fitted to RES cumulative release data [47]. The highest value of the determination coefficient (*R*^2^) of the linear regression models, which explains how well the model explains the variability of the data, was considered in order to determine which model better fitted the release data. The *R*^2^ values were 0.9848 (zero-order model), 0.9589 (first-order model), 0.9304 (Higuchi model), 0.9682 (Hixson–Crowell model) and 0.9319 (Korsmeyer–peppas model), as observable in Figures S5–S9 (SI) and in Figure 9.

Based on these results, it can be argued that the RES release followed zero-order kinetics, which means that the release of RES takes place at a constant rate, and it is independent of the RES concentration [48].

### 3.7. Antioxidant Activity of RES

The antioxidant properties of RES play an important role in its therapeutic activity. Hence, it is crucial to verify that the formulation of RES in micellar systems does not interfere with its inherent activity. The antioxidant capacity of RES was determined via the in vitro DPPH assay. This test exploits the reduction in the concentration of the stable nitrogen-centered free radical DPPH, as a measure of the free radical scavenging potential of RES. The radical is reduced in the presence of an antioxidant molecule, which captures the odd electron of DPPH, becoming paired with hydrogen, and the solution is decolored to an extent that is proportional to the decrease in the absorbance. The antioxidant activity of RES (both as raw powder and as lyophilized raw powder) was evaluated and compared with lyophilized RES-TPGS formulations. As shown in Table 3, the lyophilization process did not impact the antioxidant activity of RES, and the RSA% was comparable to that of RES raw powder. Furthermore, even the RES encapsulation (RES:TPGS formulations) did not cause a decrease in antioxidant activity. Indeed, the incorporation of the drug in the micelles did not interfere with such activity, regardless of the formulation composition. As expected, empty TPGS micelles did not show any antioxidant activity, as the proton (hydrogen) transfer to radical species by the tocopherol moiety cannot occur due to the presence of the ester bond with the succinic linker.

### 3.8. RES-TPGSs’ Effects on HaCaT Cells

**Figure 10:**
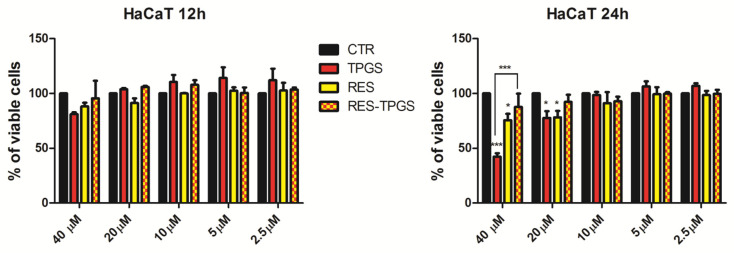
Viability of human keratinocyte HaCaT cells treated with increasing concentrations of RES, RES-TPGS 1:4 lyophilized powder, providing the same raw RES concentration, and with the TPGS concentration (2.01 μM, 4.02 μM, 8.04 μM, 16.07 μM, 32.15 μM) according to the sample DL%. The experiments were carried out three times and data are represented as the average ± S.D. * *p* < 0.05, *** *p* < 0.001.

HaCaT cells, a long-lived, spontaneously immortalized human keratinocyte line, are a suitable model for inflammatory studies, and have been extensively used for predicting surfactant toxicity [49]. Toxicity profiling is important when a new formulation is developed. Delivery systems are usually tested on cancer cells for their efficacy, ignoring the effects on normal cells. However, the safety of nanoformulations is an issue worthy of more attention. Micelles are internalized by endocytosis, but most of the time they can also undergo disassembly in the plasma membrane with the release of the drug and unimers outside the cell [29]. When dealing with non-ionic surfactants, the toxicity levels may be significantly different, depending on their chemical structure. In general, the effect of amphiphilic molecules on cells is related to their capability of interacting with the plasma membrane, leading at higher concentration to its disruption. It has been reported that surfactant toxicity decreases with hydrophilic chain elongation, as it limits the penetration into the lipid bilayer, preserving cell integrity. Indeed, poly(ethyleneglycol)-distearoyl-sn-glycero-phosphoethanolamine (PEG-DSPE, MW 2800) significantly decreased human umbilical vein Eahy.926 cell line viability at 30 µM after 24 h [50]; similarly, TPGS in our cell model triggered toxicity effects at about 32 µM (*p* < 0.001 vs. Ctr) (Figure 10).

Intriguingly, TPGS at concentrations below 10 μM slightly increased cell viability compared to control cells at both 12 and 24 h. RES encapsulation strongly limited the surfactant toxicity at higher concentrations (*p* < 0.001 vs. RES-TPGSs). It is noteworthy that only RES-TPGSs never exerted any effect on cell viability, whereas both RES and (to a great extent) TPGS triggered some toxic actions at higher doses after 24 h. In a recent study, RES induced a decrease in a concentration-dependent manner of HaCaT viability, detected by comparing different assays: MTT, neutral red uptake (NR), Alamar Blue^®^, and trypan blue. The IC_50_ values for RES varied between 174 and 412 µm, demonstrating a significant variability among the different tests employed [51]. This could depend on the different cell-death mechanism evaluated in each assay: metabolic activity (Alamar Blue^®^, MTT), cell membrane damage (trypan blue), and lysosomal integrity (NR). Our test is a DNA-based assay that has proved to be among the most sensitive cell health indicators. Our results are in concordance with those obtained with NR, which demonstrated a significant reduction of cell viability after 24 h with 25 µm of RES [51]. The toxicity data agree with stability studies and release studies. Indeed, the intact micelles can provide a sustained slow release of RES, thus reducing the toxicity of both ingredients at 24 h. Indeed, loaded micelles are probably endowed with more stability as the drug cargo inside the core could reduce the unimer release, thus decreasing TPGS interactions with the lipid bilayer. To confirm this hypothesis, our future studies will focus on micelle internalization mechanisms by preparing FITC RES-TPGS micelles. Indeed, to date, it is not clear whether TPGS is hydrolyzed to free tocopherol on the surface of the cells, or if the entire micelles are taken up by cells. In an in vitro study, it seems that tocopherol was released after it entered the cells [52]. A secondary mechanism of action may be the capability of TPGS to promote ROS generation. Similarly to α-tocopheryl succinate, TPGS can induce cancer cell apoptosis through the destruction and inhibition of mitochondrial respiratory complex II. The subsequent electron transfer chain disruption and ROS enhancement trigger cell destruction. In this case, the presence of RES could limit the DNA damage and the oxidation of lipids, proteins, and enzymes, exerting a protective role. However, this ROS-mediated pathway has been proven on tumor cells, which are more sensitive to ROS than normal cells [52].

## 4. Conclusions

There is a great deal of interest in developing novel therapies to reduce inflammation and fibrosis in pediatric as well as adult cholestatic diseases. High doses of corticosteroids represent the first-line treatment, and new gene-based strategies are currently under investigation but are still far from being usable in the near future. The importance of achieving and maintaining adequate nutritional status is of primary importance for patient health and survival after liver transplantation. For this purpose, we tried to provide a new formulation that was able to decrease malnourishment in children by embedding into TPGS an EFSA-approved vitamin E source in chronic liver diseases, RES, which has been proven to attenuate inflammation in the liver. The formulations here described were able to improve RES water solubility via micellization, without affecting its antioxidant capability. The drug release from these colloidal dispersions was sustained and constant over time, suggesting that the drug is retained inside the micelles. The effects of RES-TPGSs on the viability of normal cells demonstrated the significant reduction of TPGS toxicity, demonstrating that RES in the encapsulated form may exert more beneficial effects and, at the same time, may increase micellar stability, thus decreasing surfactant unimer interactions with the cell membrane. Hence, based on our results, we can conclude that TPGS, as a host of poorly soluble compounds, ameliorated the water solubility of RES and reduced its intrinsic cytotoxic effects as well. We are aware that the promising in vitro results reported herein could not be completely confirmed by in vivo experiments. The main limitations of our formulations could be the several additional factors that could influence the stability and RES release profiles, when administered in vivo. Therefore, future studies are necessary and are currently ongoing to demonstrate micelles’ mechanisms of internalization and the in vivo effects of TPGS-RES supplementation on rats treated with α-naphthylisothiocyanate, a well-characterized cholestatic agent.

## Figures and Tables

**Table 1:** Encapsulation efficiency (EE%) and drug loading capacity (DL%) of different RES-TPGS formulations. Each value represents the mean ± standard deviation (±SD) (degrees of freedom = 6).

RES:TPGS (w:w) Ratio in the Preparative Mixture	EE%	DL%
1:1	10 ± 1 *	9 ± 1
1:2	20 ± 2	10 ± 2
1:3	31 ± 3	11 ± 1
1:4	44 ± 5	11 ± 2
1:5	56 ± 6	11 ± 3
1:6	76 ± 1 *	13 ± 1
1:7	92 ± 6 *	15 ± 2 *

* Indicates a statistical difference between the sample mean and that of sample population (*p* < 0.05).

**Table 2:** Mean diameter (size, nm) and PDI of freshly prepared empty and loaded micelles and of formulations reconstituted from lyophilized powder, RES-TPGS formulations. The zeta potential (ƺ) values were detected on fresh RES-TPGS micelles in water. Each value represents the mean ± SD (degrees of freedom = 3).

RES:TPGS (w:w)	Size (nm) Fresh Empty	PDI Fresh Empty	Size (nm) Fresh Loaded	PDI Fresh Loaded	ƺ (mV) Loaded	Size (nm) Lyophilized	PDI Lyophilized
1:1	14.1 ± 0.6	0.27 ± 0.05	12.5 ± 0.2	0.26 ± 0.04 *	−2.2 ± 3.6	12.2 ± 0.3	0.35 ± 0.06 *
1:2	20.1 ± 0.7 *	0.20 ± 0.02	12.7 ± 0.8	0.24 ± 0.01	−3.6 ± 4.13	13.1 ± 0.6	0.27 ± 0.02
1:3	14.7 ± 0.1	0.31 ± 0.09 *	12.0 ± 0.3	0.21 ± 0.07	−3.1 ± 3.3	14.0 ± 0.7	0.32 ± 0.04
1:4	13.2 ± 0.3	0.26 ± 0.03	11.9 ± 0.8	0.19 ± 0.04	−4.1 ± 2.4	15.0 ± 0.3 *	0.212 ± 0.001 *
1:5	12.3 ± 0.5	0.10 ± 0.02 *	11.9 ± 0.2	0.24 ± 0.05	−4.6 ± 7.7 *	13.3 ± 0.4	0.298 ± 0.009
1:6	12.3 ± 0.2	0.126 ± 0.001 *	9.13 ± 0.01 *	0.13 ± 0.01 *	−4.8 ± 3.0 *	9.7 ± 0.8 *	0.28 ± 0.04
1:7	13.2 ± 0.4	0.21 ± 0.04	9.6 ± 0.3 *	0.148 ± 0.001 *	−1.6 ± 3.9 *	11.9 ± 0.1	0.245 ± 0.001

* Indicates statistical difference between the sample mean and that of the sample population (*p* < 0.05).

**Table 3:** In vitro antioxidant activity of RES (raw powder, lyophilized raw powder), RES:TPGS formulations, and TPGS alone, calculated as percentages of radical scavenging activity (RSA%). Results are expressed as mean ± S.D.

Sample	RSA%
RES pristine powder	16.37 ± 0.28
Freeze-dried RES pristine powder	15.71 ± 0.63
RES:TPGS 1:2	15.93 ± 0.02
RES:TPGS 1:6	17.96 ± 0.26
TPGS	1.24 ± 0.33

## Data Availability

All data concerning this study are contained in the present manuscript or in previous articles, the references of which have been provided.

## Supplementary Materials

The following are available online at https://www.mdpi.com/article/10.3390/pharmaceutics13081128/s1, Figure S1. RES calibration curve; Figure S2. Representative size distribution of freshly prepared RES-TPGS formulations; Figure S3. Representative size distribution of RES-TPGS formulations reconstituted from lyophilized powder; Figure S4. Representative distribution of the Z-potentials of RES-TPGS formulations; Figure S5. Zero-order kinetic mathematical model; Figure S6. First-order kinetic mathematical model; Figure S7. Higuchi kinetic mathematical model; Figure S8. Korsmayer–peppas kinetic mathematical model; Figure S9. Hixson–Crowell kinetic mathematical model; Table S1. Particle size ratio (*S_l_*/*S_f_*) of the loaded formulations after reconstitution, compared with those after fresh preparation.
