# Supplementary material for: Increased Water-Solubility and Maintained Antioxidant Power of Resveratrol by Its Encapsulation in Vitamin E TPGS Micelles: A Potential Nutritional Supplement for Chronic Liver Disease
Source: Pharmaceutics. 2021 Jul 23;13(8):1128. doi: 10.3390/pharmaceutics13081128 (PMC8400607; doi:10.3390/pharmaceutics13081128)
Supplement: Supplementary file 1 [file pharmaceutics-13-01128-s001.zip › pharmaceutics-1300382-supplementary.pdf]

pharmaceutics

MDPI

Supplementary Materials: Increased Water-Solubility and Maintained Antioxidant Power of Resveratrol by Its Encapsulation in Vitamin E TPGS Micelles: A Potential Nutritional Supplement for Chronic Liver Disease

Guendalina Zuccari, Silvana Alfei, Alessia Zorzoli, Danilo Marimpietri, Federica Turrini, Sara Baldassari, Leonardo Marchitto and Gabriele Caviglioli

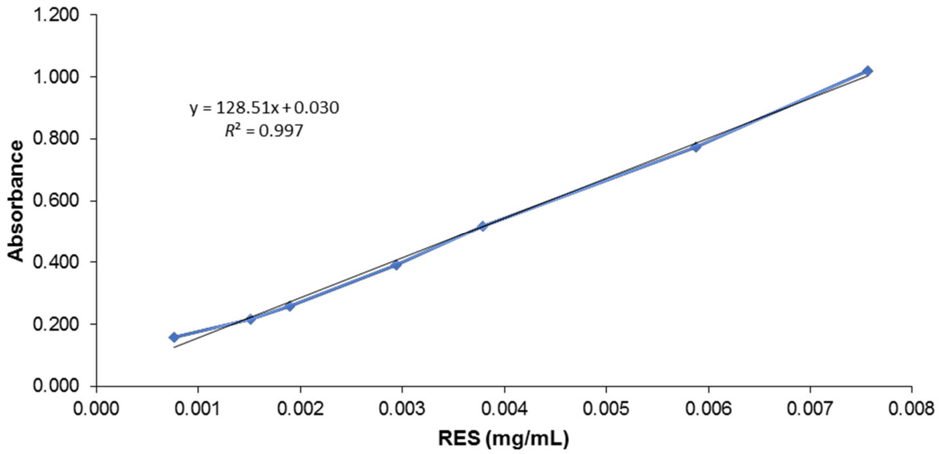

Figure S1. RES calibration curve in methanol at λ_{max} = 306 nm using an UV-Vis spectrophotometer within a range 3.33–331.70 μM.

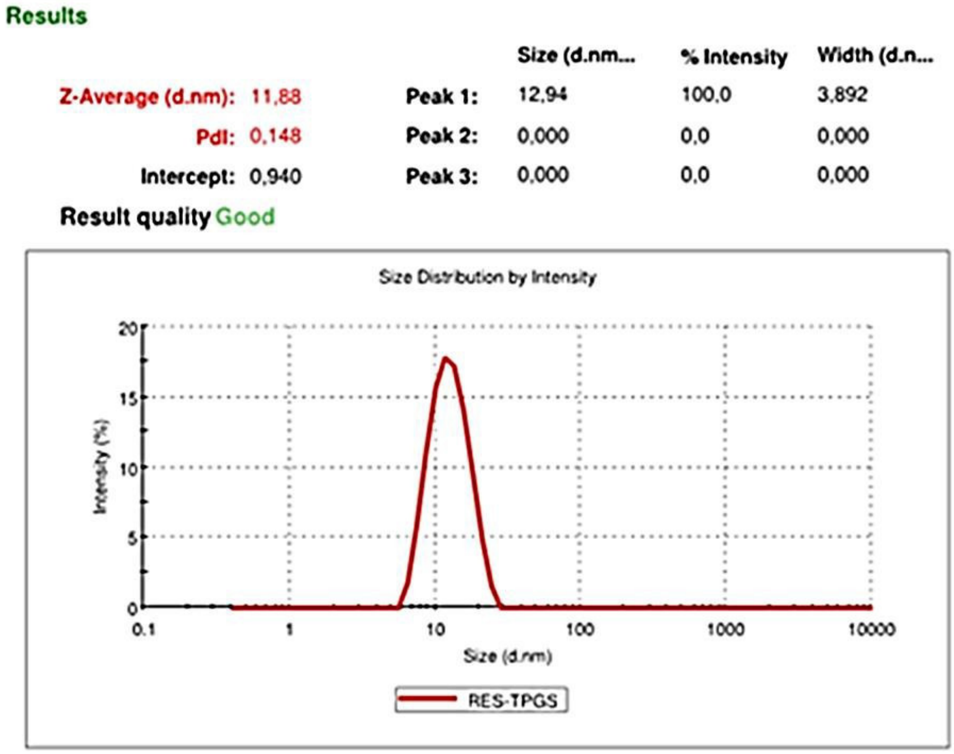

Figure S2. Representative size distribution of freshly prepared RES-TPGS colloidal dispersion.

Results

			Size (d.nm...	% Intensity	Width (d.n...
Z-Average (d.nm):	11,03	Peak 1:	12,13	100,0	3,273
PdI:	0,110	Peak 2:	0,000	0,0	0,000
Intercept:	0,940	Peak 3:	0,000	0,0	0,000

Result quality Good

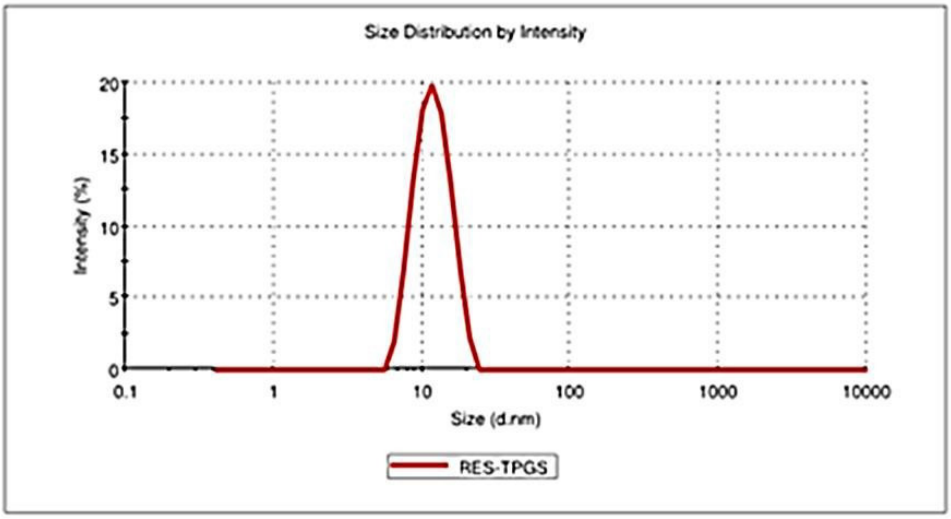

Figure S3. Representative size distribution of a RES-TPGS formulation reconstituted in water to its original volume from lyophilized powder.

Results

			Mean (mV)	Area (%)	Width (mV)
Zeta Potential (mV):	-4,75	Peak 1:	-4,75	100,0	3,04
Zeta Deviation (mV):	3,04	Peak 2:	0,00	0,0	0,00
Conductivity (mS/cm):	0,00600	Peak 3:	0,00	0,0	0,00

Result quality Good

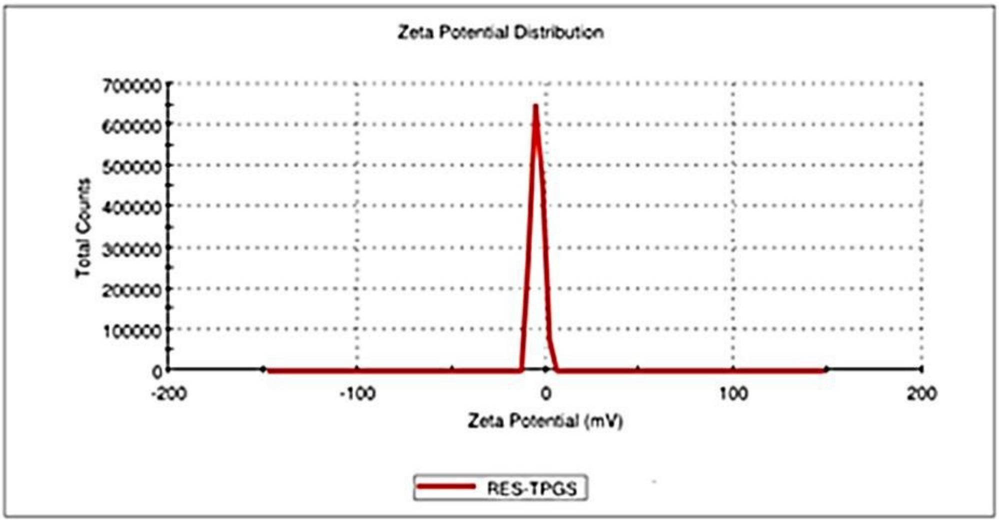

Figure S4. Representative distribution of the Z potentials of a RES-TPGS formulation measured in water.

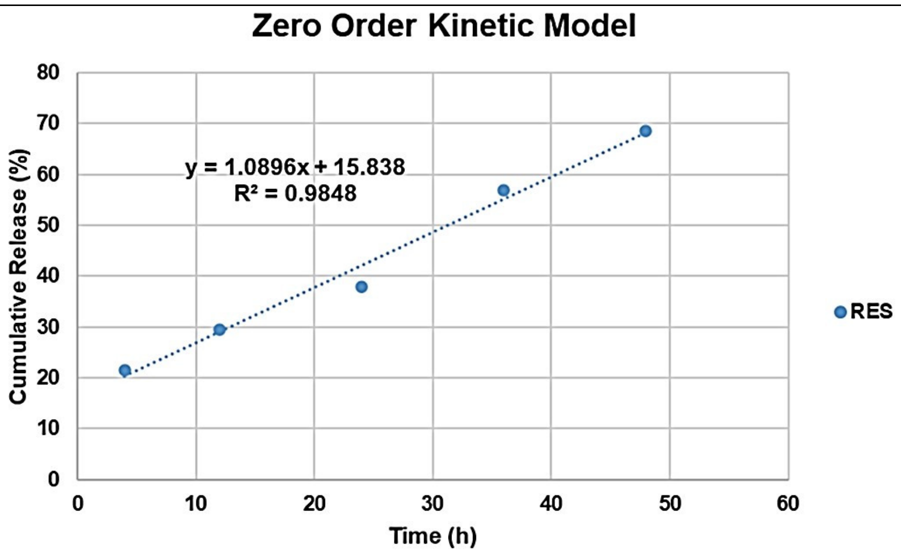

Figure S5. Zero order kinetic mathematical model.

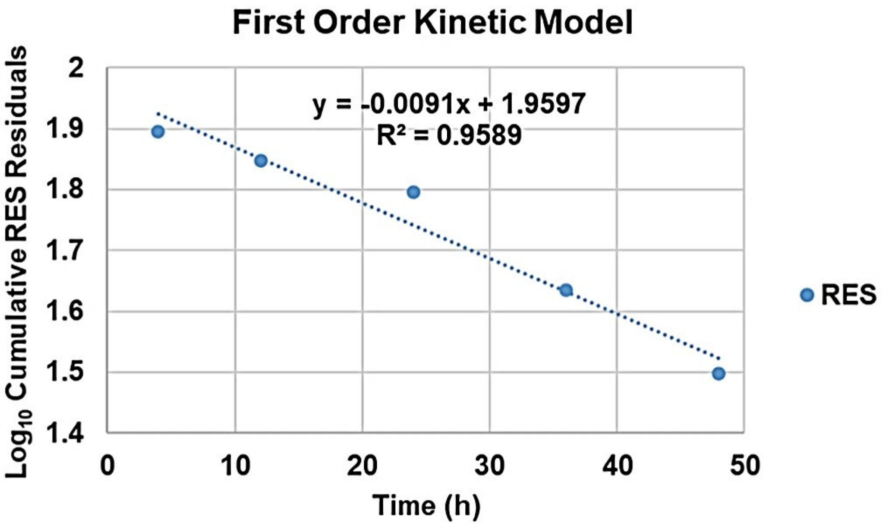

Figure S6. First order kinetic mathematical model.

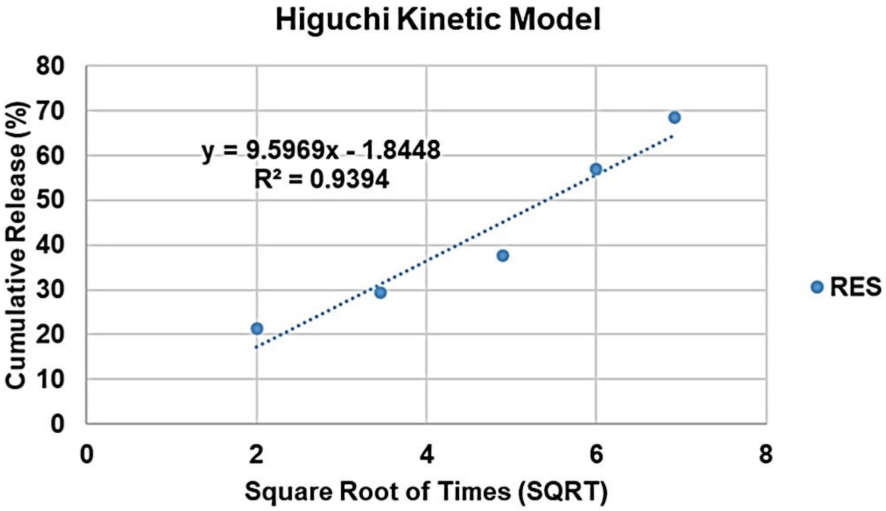

Figure S7. Higuchi kinetic mathematical model.

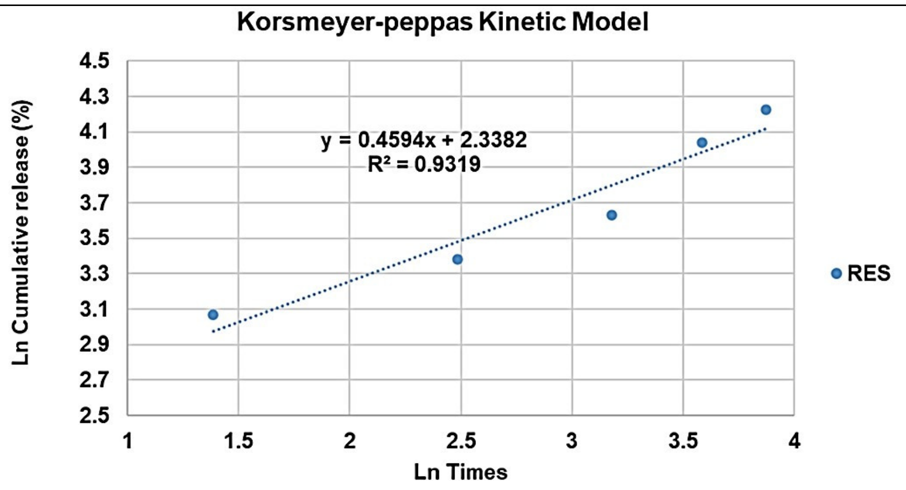

Figure S8. Korsmayer-Peppas kinetic mathematical model.

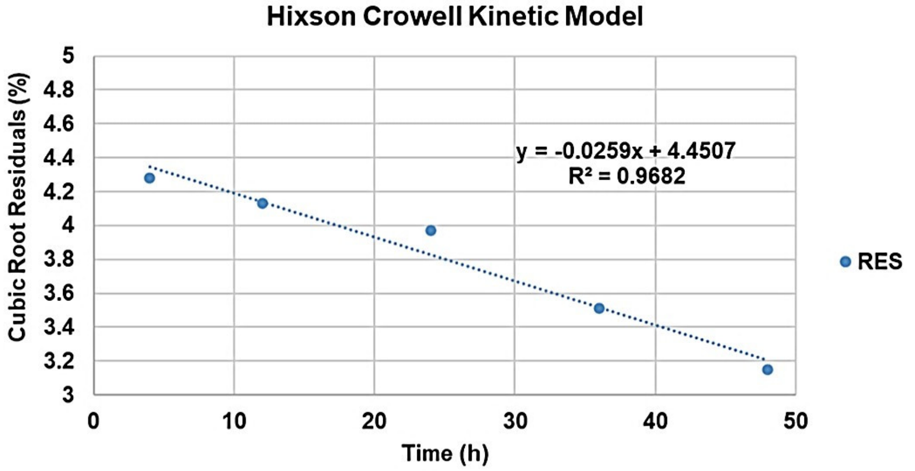

Figure S9. Hixson Crowel kinetic mathematical model.

Table S1. Particle size ratio (*Sl*/*Sf*) of the loaded formulations after recostitution compared with those after fresh preparation.

TPGS Concentration (mg/mL)	*Sl*/*Sf* ratio [1]
2	1.0± 0.4
4	1.0± 0.2
6	1.2±0.1
8	1.3±0.4
10	1.1±0.2
12	1.1±0.1
14	1.2±0.3

[1] Mean ± S.D. (*n*=3).
